# *pheS*^*AG*^ Based Rapid and Efficient Markerless Mutagenesis in *Methylotuvimicrobium*

**DOI:** 10.3389/fmicb.2020.00441

**Published:** 2020-03-31

**Authors:** Yongchuang Liu, Xiangrong He, Pingping Zhu, Minggen Cheng, Qing Hong, Xin Yan

**Affiliations:** ^1^Key Laboratory of Agricultural Environmental Microbiology, Ministry of Agriculture, College of Life Sciences, Nanjing Agricultural University, Nanjing, China; ^2^Jiangsu Provincial Key Lab for Organic Solid Waste Utilization, Nanjing Agricultural University, Nanjing, China

**Keywords:** counterselection, *pheS*, *Methylotuvimicrobium*, electroporation-based, markerless mutagenesis

## Abstract

Due to their fast growth rate and robustness, some haloalkalitolerant methanotrophs from the genus *Methylotuvimicrobium* have recently become not only promising biocatalysts for methane conversion but also favorable materials for obtaining fundamental knowledge on methanotrophs. Here, to realize unmarked genome modification in *Methylotuvimicrobium* bacteria, a counterselectable marker (CSM) was developed based on *pheS*, which encodes the α-subunit of phenylalanyl-tRNA synthetase. Two-point mutations (T252A and A306G) were introduced into PheS in *Methylotuvimicrobium buryatense* 5GB1C, generating PheS^*AG*^, which can recognize *p*-chloro-phenylalanine (*p*-Cl-Phe) as a substrate. Theoretically, the expression of PheS^*AG*^ in a cell will result in the incorporation of *p*-Cl-Phe into proteins, leading to cell death. The P*_*tac*_* promoter and the ribosome-binding site region of *mmoX* were employed to control *pheS*^*AG*^, producing the *pheS^*AG*^-3* CSM. *M. buryatense* 5GB1C harboring *pheS^*AG*^-3* was extremely sensitive to 0.5 mM *p*-Cl-Phe. Then, a positive and counterselection cassette, PZ (only 1.5 kb in length), was constructed by combining *pheS^*AG*^-3* and the zeocin resistance gene. A PZ- and PCR-based strategy was used to create the unmarked deletion of *glgA1* or the whole *smmo* operon in *M. buryatense* 5GB1C and *Methylotuvimicrobium alcaliphilum* 20Z. The positive rates were over 92%, and the process could be accomplished in as few as eight days.

## Introduction

Methane, the principal component of natural gas and biogas, is a major candidate source of carbon for (bio)chemical synthesis (Hanson and Hanson, 1996), and the conversion of methane into valuable products has been pursued off and on for almost half a century (Strong et al., 2015). Methane-oxidizing bacteria (methanotrophs) are able to use methane as their sole source of carbon and energy and thus are promising systems for methane-based bioconversion (Hanson and Hanson, 1996; Fei et al., 2014). Since the 1970s, most studies and biotechnological efforts have focused on well-characterized species, such as *Methylococcus capsulatus* Bath, *Methylosinus trichosporium* OB3b, and *Methylocystis parvus* OBBP (Hou et al., 1979; Kalyuzhnaya et al., 2015). In recent years, due to their fast growth rate and robustness, some haloalkaliphilic *Methylotuvimicrobium* bacteria, such as *Methylotuvimicrobium buryatense* 5GB1C and *Methylotuvimicrobium alcaliphilum* 20Z, have been considered particularly promising methanotrophs for industrial use (Trotsenko et al., 2005; Andrea et al., 2015; Nguyen et al., 2018). For strains 5GB1C and 20Z, the genome, transcriptome, and metabolic pathway have been well characterized (Kalyuzhnaya et al., 2013; Strong et al., 2016), the genetic tools have been established (Kalyuzhnaya et al., 2015; Puri et al., 2015; Yan et al., 2016; Nguyen et al., 2018), and metabolic engineering to generate value-added products from methane has been attempted (Andrea et al., 2015; Demidenko et al., 2016; Henard et al., 2016; Nguyen et al., 2018). Promising strains and synthetic biology give new optimism for a realized methane-based bio-industry.

Markerless chromosomal modification is considered an ideal genetic manipulation with no polar effects and guaranteed safety against gene flow by evicting the resistance marker gene. The Flp/FRT site-specific recombination system and a counterselectable marker (CSM), *sacB*, have been employed to generate a markerless chromosomal modification in *Methylotuvimicrobium* (Puri et al., 2015; Yan et al., 2016; Nguyen et al., 2018). However, the Flp/FRT system leaves an FRT site at the replacement locus (Hoang et al., 1998; Kühn and Torres, 2002), which interferes with subsequent rounds of manipulations in the same host; counterselection based on *sacB* and sucrose does not leave a scar on the chromosome, but the positive rate is typically 5–50% in *Methylotuvimicrobium* according to previous reports (Yan et al., 2016) and our experience. Therefore, an efficient CSM is still needed in *Methylotuvimicrobium*.

The *pheS* gene encodes the α-subunit of phenylalanyl-tRNA synthetase, which is highly conserved among bacteria. PheS with T251A and A294G substitutions (PheS^*AG*^) has the ability to aminoacylate the phenylalanine analog *p*-chloro-phenylalanine (*p*-Cl-Phe) in *Escherichia coli* (Kast and Hennecke, 1991; Kentaro, 2015). Incorporation of *p*-Cl-Phe into proteins lead to cell death (Kast and Hennecke, 1991; Kast, 1994). Therefore, *pheS*^*AG*^ has been used as a CSM in various bacteria for marker-free genome modification (Kristich et al., 2007; Barrett et al., 2008; Zhoujie et al., 2011; Carr et al., 2015; Xin et al., 2017; Ishikawa et al., 2018). This work aimed to develop *pheS* as an efficient CSM for *Methylotuvimicrobium* and to establish a fast marker-free genome modification method for these industrially promising methanotrophs.

## Materials and Methods

### Bacterial Strains and Culture Conditions

All strains were cultured in an atmosphere of 25% methane in air at 30°C. *M. buryatense* 5GB1C and its derived strains were grown in NMS2 medium (Puri et al., 2015). *M. alcaliphilum* 20Z and its derived strains were grown in NMS3 medium (Khmelenina et al., 1997). Plates were incubated in sealed jars (Oxoid Limited, Hampshire, United Kingdom), while liquid cultures were grown in 100 ml glass serum bottles sealed with rubber stoppers and aluminum seals. The total of 30 μg/ml zeocin (Zeo) was added if required.

### DNA Manipulation Techniques

Oligonucleotide synthesis (listed in Supplementary Table S1) and DNA sequencing were performed by Sangon Biotech Co., Ltd. (Shanghai, China). The isolation and manipulation of DNA were carried out using standard techniques. All enzymes were commercial preparations and were used as specified by the supplier (NEB, Shanghai, China).

### Fusion of Multiple DNA Fragments by Overlap PCR

Fusion of multiple DNA fragments by overlap PCR was carried out as described by Shevchuk et al. (2004). In brief, overlaps of approximately 30–40 nucleotides were introduced between each of 2 fragments through primers. The reaction mixture of step A contained 11.5 μl of water, 4 μl of Phusion buffer (×5), 2 μl of deoxynucleoside triphosphate (dNTP) mix (2.5 mM each), 2 μl of gel-purified fragments (approximately 100 ng each), and 0.5 μl of Phusion DNA polymerase. The cycling parameters were an initial denaturation at 98°C for 3 min and subsequent steps of 98°C for 15 s, annealing at 55°C for 10 s and extension at 72°C for 3 min for 15 cycles total. The reaction mixture of step B contained 34.5 μl of water, 10 μl of Phusion buffer, 4 μl of dNTPmix, 2 μl of forward and reverse primers (10 mM) specific for the expected fragment, and 1 μl of the unpurified PCR product from step A and 0.5 μl of Phusion DNA polymerase. The cycling parameters were an initial denaturation at 98°C for 2 min and subsequent steps of 98°C for 10 s, annealing at 58°C for 10 s, and extension at 72°C for 3 min for 30 cycles total.

### Construction of *pheS*^*AG*^

The site-directed mutations in *pheS*^*AG*^ (ACC252GCA) and (GCA306GGT) were generated by overlap extension with phes1F/phes1R, phes2F/phes2R, and phes3F/phes3R primer pairs using the genomic DNA of *M. buryatense* 5GB1C as a template. The ACC252GCA mutation was introduced by the primer phes1R and the GCA306GGT mutation was introduced by the primer phes3F. The *pheS*^*AG*^ mutant was named *pheS^*AG*^-1*. The expression cassette *pheS^*AG*^-2* was constructed based on *pheS^*AG*^-1*. The promoter region of *pheS^*AG*^-1* was replaced by P*_*tac*_* with the ZPtac1F/zeoR primer pair, and P*_*tac*_* was introduced by ZPtac1F. The ribosome-binding site (RBS) region of *pheS^*AG*^-2* was replaced by the RBS region of *mmoX* using the ZPtac2F/zeoR primer pair, thereby generating *pheS^*AG*^-3.*

### p-Cl-Phe Sensitivity Assessment

Expression cassettes *pheS^*AG*^-1*, *pheS^*AG*^-2*, and *pheS^*AG*^-3* were individually inserted at chromosome loci between the genes METBUDRAFT_2794 and METBUDRAFT_2795 in strain 5GB1C. The insertion construct containing each cassette and the flanking regions were assembled using PCR with the primers indicated in Supplementary Table S1. The assembled products were transformed into *M. buryatense* 5GB1C by the electroporation method (Yan et al., 2016). To assess *p*-Cl-Phe sensitivity, the bacterial strain was grown in NMS2 or NMS3 medium with the corresponding antibiotics with OD_600_ = 1.0, then the cell cultures were serially diluted 1:10; a sample from each serial dilution was spotted onto agar plates containing *p*-Cl-Phe at different concentrations, and the plates were incubated at 30°C with methane vapor for 5 days.

### Construction of the PZ Cassette

The PZ cassette was constructed in two steps using overlap PCR. First, the *zeo* gene, together with its RBS sequence, from 5GB1C-Ppmo-xylE was amplified with zeoF/zeoR primer pair. Then, the *zeo* gene was fused to the 3′ end of *pheS^*AG*^-3* with phes1F and zeoR primers. Thus, *pheS^*AG*^-3* and *zeo* were arranged into an operon under the control of P*_*tac*_* and RBS*_*mmoX*_*.

### Construction of Mutants With *glgA1* Deleted in *M. buryatense* 5GB1C

To generate the *glgA1* deletion mutation in strain 5GB1C, the left flanking (LF) region (∼800 bp), direct repeat (DR) sequence (∼450 bp), and right flanking (RF) region (∼800 bp) from strain 5GB1C were amplified using the gA1LF-F/gA1LF-R, gA1DR-F/gA1DR-R, and gA1RF-F/gA1RF-R primer pairs, respectively. The PZ cassette was amplified with the gA1PZ-F/gA1PZ-R primer pair using *pheS^*AG*^-3* as a template. These four fragments were fused using overlap PCR in the order of the LF, DR, PZ cassette, and RF. The resulting ∼3.5-kb *glgA1* deletion amplicon (PCR product) was directly transformed into strain 5GB1C by the electroporation method (Yan et al., 2016). The DR and PZ cassette were inserted immediately upstream of the target region via a double-crossover recombination event without any deletions, which was selected by Zeo^*r*^ (for approximately 3 days). The transformants were transferred to NMS2 plates and maintained overnight at 30°C. Then, the cells were transferred to 5 ml of NMS2 medium and cultivated for approximately 10 h at 30°C to an OD_600_ = 1.0. Then, 100 μl of cells were spread onto selective plates (for approximately 3–4 days). Mutants growing on selective plates were further confirmed by PCR and DNA sequencing.

### Deletion of the 10-kb *smmo* Operon in *M. buryatense* 5GB1C

Deletion of the *smmo* operon was carried out using the same strategy as described for *glgA1* deletion. The LF, DR, PZ cassette, and RF fragments were amplified using the MOLF-F/MOLF-R, MODR-F/MODR-R, MOPZ-F/MOPZ-R, and MORF-F/MORF-R primer pairs, respectively. The four fragments were fused to generate the ∼3.5-kb *smmo* deletion amplicon, which was subsequently transformed into strain 5GB1C.

### Construction of the PZ^∗^ Cassette

The PZ^∗^ cassette was constructed based on *pheS^*AG*^-3*. We engineered a series of silent mutations in *pheS^*AG*^-3* to reduce its similarity to wild-type *pheS*. The artificially synthesized *pheS*, including a *tac* promoter, RBS of *mmoX* (RBS*_*mmoX*_*), and mutated *pheS* (*pheS*^*AG*^), was fused to the *zeo* gene to obtain the PZ^∗^ cassette using the PZ^∗^LF-F/PZ^∗^LF-R and PZ^∗^RF-F/PZ^∗^RF-R primer pairs.

### Deletion of *glgA1* in *M. alcaliphilum* 20Z

The LF region (∼800 bp), DR sequence (∼450 bp), and RF region (∼800 bp) from strain 20Z were amplified using the gALF-F/gALF-R, gADR-F/gADR-R, and gARF-F/gARF-R primer pairs, respectively. The PZ^∗^ cassette was amplified with the gAPZ-F/gAPZ-R primer pair. These four fragments were fused to generate the ∼3.5-kb *glgA1*-deletion amplicon and were directly transformed into strain 20Z by electroporation.

### Naphthalene Assay

To detect the activity of soluble methane monooxygenase (sMMO), *M. buryatense* strains were grown in an NMS2 medium without copper at 30°C. A naphthalene oxidation assay was routinely used for the qualitative detection of sMMO activity (Graham et al., 1992). Approximately 50 mg of crushed naphthalene crystals were added to a 3 ml batch culture of *M. buryatense* and the mixture was shaken at 30°C for 2 h. The cell suspension was then centrifuged for 2 min at 12,000 *g*. A total of 50 μl of 5 mg ml^–1^ freshly prepared tetrazotized *o*-dianisidine was added to the supernatant. The deep purple color of the mixture indicated sMMO activity.

## Results

### p-Cl-Phe Sensitivity of *M. buryatense* 5GB1C and *M. alcaliphilum* 20Z

**FIGURE 1:**
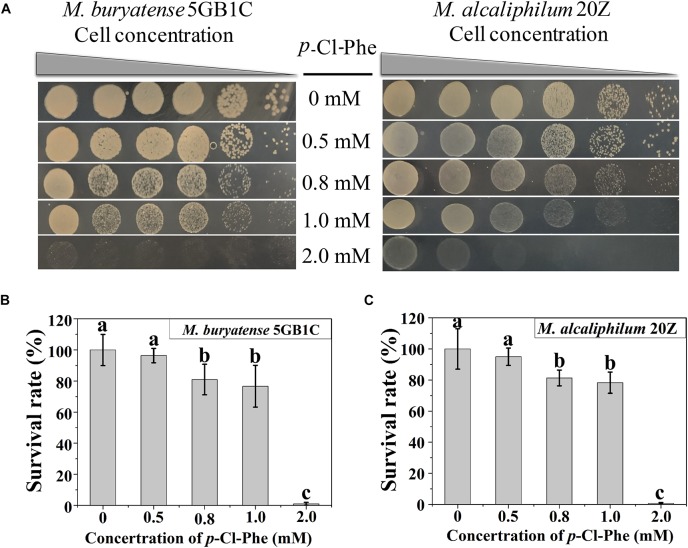
*p*-Cl-Phe sensitivity of *M. buryatense* 5GB1C and *M. alcaliphilum* 20Z. **(A)** Cell cultures were serially diluted 1:10. Each serial dilution was spotted onto agar plates containing *p*-Cl-Phe at different concentration. Plates were incubated in sealed jars at 30°C in an atmosphere of 25% methane in air for 5 days. **(B,C)** Cells were grown to an OD_600_ of 1.0, diluted 10^4^-fold and plated on NMS2 (*M. buryatense* 5GB1C) or NMS3 (*M. alcaliphilum* 20Z) medium containing different concentrations of *p*-Cl-Phe. All data were expressed as means ± standard deviations (SD) (*n* = 3). Letters above bars indicate significant differences (*P* < 0.05). The survival rate of each strain on NMS2 or NMS3 plate was defined as 100%.

To determine whether *p*-Cl-Phe could be used for counterselection with its marker *pheS*^*AG*^ in *M. buryatense* 5GB1C and *M. alcaliphilum* 20Z, the resistance of both strains to different concentrations of *p*-Cl-Phe was tested. As shown in Figure 1, strains 5GB1C and 20Z exhibited similar sensitivity to *p*-Cl-Phe. The growth of both strains was slightly inhibited in the presence of 0.5 mM *p*-Cl-Phe (with survival rates of 96 and 95%), moderately inhibited at 0.8 or 1.0 mM *p*-Cl-Phe (with survival rates of 81 to 75%), and greatly inhibited with the addition of 2 mM *p*-Cl-Phe (with survival rates less than 1%). These results indicate that the concentration of *p*-Cl-Phe to be added during counterselection should be less than 1 mM.

### Optimizing the Expression Level of *pheS*^*AG*^

**FIGURE 2:**
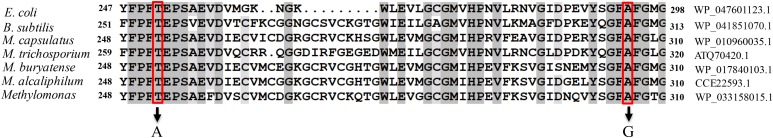
Sequence alignment of PheS. The PheS C-terminal region of strains *Escherichia coli* K12, *Bacillus subtilis* 168, *M. capsulatus* (Bath), *M. trichosporium* OB3b, *M. buryatense* 5GB1C, *M. alcaliphilum* 20Z, and *Methylomonas* sp. LW13 were aligned using ClustalW2. The residues boxed with a red line indicates the conserved alanine residue that can be subjected to mutagenesis to create *p*-Cl-Phe sensitivity.

To identify the amino acid residues for mutagenesis, multiple sequence alignment of the PheS proteins from various species was carried out using ClustalW2 (Larkin et al., 2007). As shown in Figure 2, the two residues of T252 and A306 in PheS*_*Mb*_* or PheS*_*Ma*_* corresponded to T251 and A294 in PheS*_*Ec*_*, respectively. Therefore, two substitutions of T252A and A306G were introduced into PheS*_*Mb*_* through point mutation, generating PheS^*AG*^, which would theoretically enhance the *p*-Cl-Phe sensitivity of *M. buryatense* 5GB1C. In addition, PheS*_*Mb*_* had 91.5% sequence identity with PheS*_*Ma*_*, suggesting that they can likely replace each other.

**FIGURE 3:**
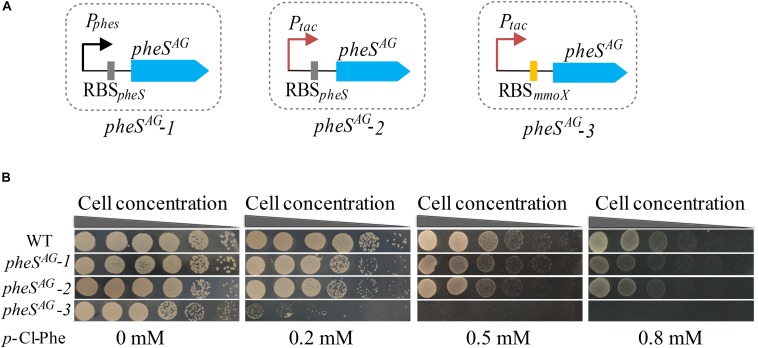
Optimizing the expression level of *pheS*^*AG*^ in *M. buryatense* 5GB1C. **(A)** Different promoters and RBS were used to control the expression of *pheS*^*AG*^. *P_*phe*__*S*_* and RBS*_*pheS*_*, the promoter and ribosome-binding site (RBS) region of wild type *pheS*; *P*_*tac*_, tac promoter; RBS*_*mmoX*_*, the RBS region of *mmoX*. **(B)** *p*-Cl-Phe sensitivity of mutants containing different *pheS*^*AG*^. The sensitivity of 5G (*pheS^*AG*^-1*) and 5G (*pheS^*AG*^-2*) to *p*-Cl-Phe was not significantly different from that of wild type while the 5G (*pheS^*AG*^-3*) mutant was extremely sensitive to *p*-Cl-Phe.

To test whether the expression of PheS^*AG*^ enhanced the sensitivity of *M. buryatense* 5GB1C toward *p*-Cl-Phe, *pheS^*AG*^-1* (*pheS*^*AG*^ with its native transcription and translation signals) (Figure 3A) was inserted between the METBUDRAFT_2794 and METBUDRAFT_2795 genes in the chromosome of strain 5GB1C (Yan et al., 2016). However, strain 5GB1C containing *pheS^*AG*^-1* displayed subtly enhanced sensitivity toward *p*-Cl-Phe (Figure 3B), indicating that the expression of PheS^*AG*^ was insufficient to induce *p*-Cl-Phe sensitivity. Since P*_*tac*_* was demonstrated to be a strong promoter in *M. buryatense* 5GB1C (Puri et al., 2015; Demidenko et al., 2016), the native promoter of *pheS*^*AG*^ was replaced by P*_*tac*_*, thus generating the expression cassette *pheS^*AG*^-2* (Figure 3A). Unfortunately, cassette *pheS^*AG*^-2* also failed to induce high sensitivity against 0.5–1 mM *p*-Cl-Phe in strain 5GB1C (Figure 3B).

The RBS region is crucial to the translation rate, which is usually used to tune protein expression (Barrick et al., 1994; Teramoto et al., 2011; Ravasi et al., 2012; Shi et al., 2018; Opgenorth et al., 2019). Considering that MmoX is an abundantly expressed protein in the absence of copper (Semrau et al., 2010), its RBS region was expected to trigger efficient translation initiation. Therefore, P*_*tac*_* and RBS*_*mmoX*_* were combined to control the expression of PheS^*AG*^, generating the expression cassette *pheS^*AG*^-3* (Figure 3A). As shown in Figure 3B, *M. buryatense* 5GB1C containing *pheS^*AG*^-3* was extremely sensitive to *p*-Cl-Phe at concentrations over 0.5 mM, suggesting that *pheS^*AG*^-3* could be used as an efficient CSM in strain 5GB1C.

### pheS^*AG*^-3, Zeocin Resistance Marker and PCR Based Markerless Deletion Strategies

**FIGURE 4:**
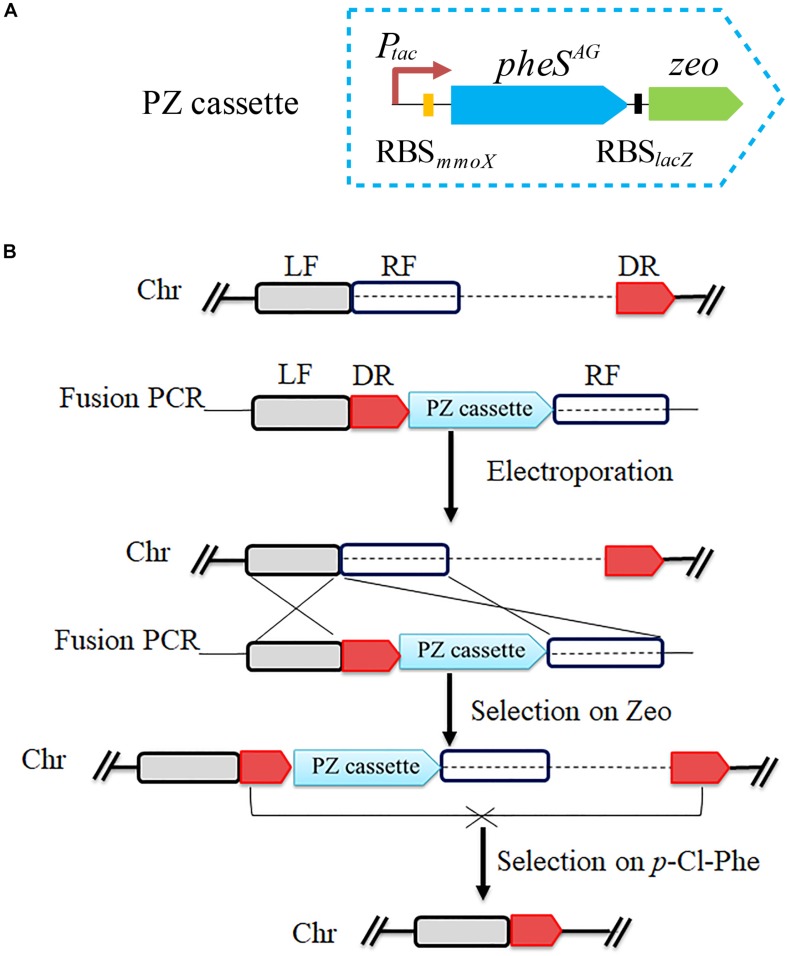
Scheme for *pheS^*AG*^-3* and PCR-based marker-free DNA fragment deletion. **(A)** Construction of PZ cassette. The *pheS^*AG*^-3* was fused with zeocin resistance gene to construct a PZ cassette. **(B)** PZ cassette and PCR based markerless deletion strategy. To delete a target fragment (dotted line), a ∼450-bp region just downstream of target fragment is used as a direct repeat sequence (DR) and is put ahead of the PZ cassette. Transformants with an insertion of the fragment containing the DR and PZ cassette just upstream of the target region are selected on zeocin. Recombination between two DR sequences excises both the PZ cassette and the target fragment, and the resulting mutant is selected on 0.8 mM *p*-Cl-Phe. *P*_*tac*_, tac promoter; *zeo*, zeocin resistance gene; LF, left flanking region; RF, right flanking region; Chr, chromosome.

To verify the feasibility of *pheS^*AG*^-3* as a CSM, a positive and counterselection cassette named PZ was constructed by assembling the SD sequence of *lacZ* and the *Sh ble* gene (*zeo*) behind *pheS^*AG*^-3* (Figure 4A). PZ is only approximately 1.5 kb long. The PZ- and PCR-based marker-free deletion was performed according to previous reports (Yan et al., 2016). Briefly, a 450-bp fragment immediately downstream of the target region to be deleted was used as the DR sequence, then, the LF and RF regions (∼800 bp on each side), DR and PZ were fused by overlapping PCR in the order of LF, DR, PZ, and RF, and the product was transferred into strain 5GB1C by electroporation. The DR and PZ were inserted immediately upstream of the target region without any deletion, which was selected by zeocin; finally, the target region together with PZ was excised via recombination between DRs and selected by 0.5 mM *p*-Cl-Phe (Figure 4B).

**FIGURE 5:**
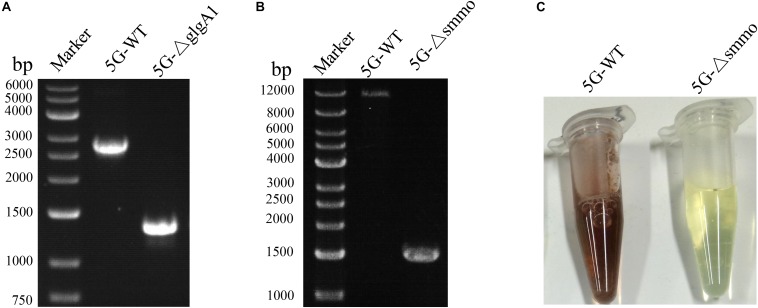
The deletion of *glgA1* and *smmo* operon in *M. buryatense* 5GB1C. **(A)** PCR confirmation of *glgA1* deletion. PCR was performed using the primers YZ1-F and YZ1-R. The nucleotide sequences of these primers are shown in Supplementary Table S1. From the genome sequence information for *M. buryatense* 5GB1C, the lengths of PCR amplicons from the wild type and the Δ*glgA1* mutant are expected to be 2715 and 1284 bp, respectively. **(B)** PCR confirmation of *smmo* deletion. PCR was performed using the primers YZ2-F and YZ2-R. The nucleotide sequences of these primers are shown in Supplementary Table S1. From the genome sequence information for *M. buryatense* 5GB1C, the lengths of PCR amplicons from the wild type and the ΔsMMO mutant are expected to be 12,199 and 1530 bp, respectively. **(C)** The sMMO activity of strains 5GB1C and 5G-Δsmmo. Cells grown in NMS2 medium without copper were subjected to naphthalene oxidation assay developed by Graham et al. (1992). The cells expressing sMMO turn deep purple.

**FIGURE 6:**
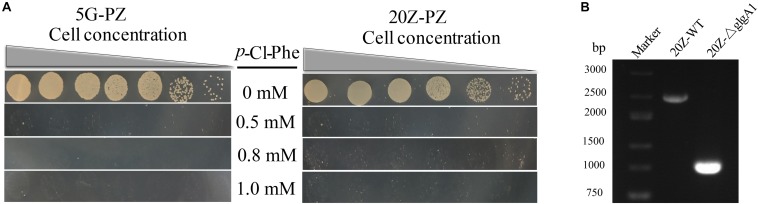
PZ cassette-based marker-free DNA fragment deletion. **(A)** *p*-Cl-Phe sensitivity of 5G-PZ and 20Z-PZ. Cell cultures were serially diluted 1:10. Each serial dilution was spotted onto agar plates containing *p*-Cl-Phe at different concentration. Plates were incubated in sealed jars at 30°C in an atmosphere of 25% methane in air for 5 days. **(B)** The deletion of *glgA1* in *M. alcaliphilum* 20Z. PCR was performed using the primers YZ3-F and YZ3-R. The nucleotide sequences of these primers are shown in Supplementary Table S1. From the genome sequence information for *M. alcaliphilum* 20Z, the lengths of PCR amplicons from the wild type and the Δ*glgA1* mutant are expected to be 2454 and 1023 bp, respectively.

Both *glgA1* (1.5 kb) and the *smmo* operon (∼10 kb) were successfully deleted using this strategy in *M. buryatense* 5GB1C (Figure 5). For each deletion, fifty colonies on the counterselection plate were verified by PCR, and the positive rates were 94 and 92% for *glgA1* and the *smmo* operon, respectively. Moreover, the whole deletion process could be completed within 8 days. Then this strategy was employed to knock out *glgA1* in *M. alcaliphilum* 20Z. Similar to *M. buryatense* 5GB1C, *M. alcaliphilum* 20Z harboring the PZ cassette was also extremely sensitive to *p*-Cl-Phe at concentrations over 0.5 mM (Figure 6A), indicating that PheS^*AG*^ of *M. buryatense* could be compiled with PheT_*M.a*_. After counterselection, fifty colonies were tested by PCR (Figure 6B), and 46 were positive, with a positive rate of 94%.

### Recoding *pheS*^*AG*^ to Avoid Homologous Recombination Between *pheS*^*AG*^ and *pheS*

**FIGURE 7:**
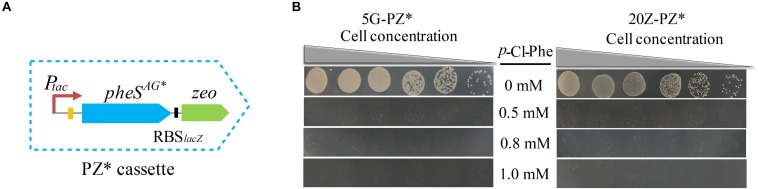
PZ* cassette-based marker-free DNA fragment deletion. **(A)** Construction of PZ* cassette. **(B)** *p*-Cl-Phe sensitivity of 5G-PZ* and 20Z-PZ*. Cell cultures were serially diluted 1:10. Each serial dilution was spotted onto agar plates containing *p*-Cl-Phe at different concentration. Plates were incubated in sealed jars at 30°C in an atmosphere of 25% methane in air for 5 days.

Since *pheS*^*AG*^ was almost identical to *pheS*, undesired homologous recombination between them may occur during genome modification, leading to false positive results. To avoid this undesired homologous recombination, we sought to decrease the similarity between them by recoding *pheS*^*AG*^. *pheS*^*AG*^ was recoded according to the codon usage biases of several highly expressed proteins, namely, particulate methane monooxygenase, sMMO, and methanol dehydrogenase. The new gene *pheS*^AG*^ shared 68% similarity to *pheS* (Supplementary Figure S1). Then *pheS*^*AG*^ in PZ was replaced by *pheS*^AG^*^, generating a new positive and counterselection cassette PZ^∗^ (Figure 7A). Similar to PZ, PZ^∗^ also conferred *M. buryatense* 5GB1C and *M. alcaliphilum* 20Z with high sensitivity to *p*-Cl-Phe at concentrations over 0.5 mM (Figure 7B). The marker-free deletion of *glgA1* was accomplished using PZ^∗^ in both strains, with positive rates over 92% (data not shown).

## Discussion

In this study, two mutations, T252A and A306G, were introduced into the PheS of *M. buryatense* 5GB1C, and the resulting PheS^*AG*^ mutant could recognize *p*-Cl-Phe. Then, strong transcription and translation signals (promoter P*_*tac*_* and RBS*_*mmoX*_*) were used to enhance the expression level of PheS^*AG*^ to the extent that the host cell failed to grow in the presence of 0.5 mM *p*-Cl-Phe, demonstrating that the *pheS^*AG*^-3* expression cassette was an effective CSM. A positive and counterselection cassette PZ^∗^ was constructed based on *pheS^*AG*^-3* and the zeocin resistance gene. A PZ^∗^- and PCR-based method enabled fast and efficient markerless genome deletion in *M. buryatense* 5GB1C and *M. alcaliphilum* 20Z, two promising methanotrophs for methane-based bioconversion. Furthermore, point mutations and foreign DNA insertions can also be easily realized using this method.

This method has several advantages over existing methods. First, no scar was left after modification (the Flp/FRT system will leave the FRT site) (Hoang et al., 1998). Second, counterselection was very efficient, with a positive rate greater than 92%. This is much higher than that typically achieved in *sacB* counterselection, which typically employs a single crossover event to integrate mutant cassettes prior to counterselection (and, in turn, often leads to <50% efficiency) (Puri et al., 2015). Third, the PZ^∗^ cassette was only 1.5 kb in length, and thus, the deletion construct could easily be assembled by PCR. The CRISPR/Cas9 system may be used with *Methylotuvimicrobium* in the future, but this system depends on vector construction and usually delivers low efficiency and off-target effects (Slaymaker et al., 2016; Chen et al., 2017; Tapscott et al., 2019). Therefore, the method developed here is a desired unmarked genetic manipulation tool for *Methylotuvimicrobium*.

This work also offers an alternative strategy to increase the expression level of PheS^*AG*^. Since the mutant PheS^*AG*^ must compete with the endogenous wild-type PheS to form complexes with PheT (Mermershtain et al., 2011), only when the mutant PheS^*AG*^ is abundantly expressed can the bacteria acquire greater sensitivity sensitive to *p*-Cl-Phe. According to a previous strategy of using strong promoters (Puri et al., 2015), the promoter P*_*tac*_* was used to increase the expression of PheS^*AG*^, but this effort failed to enhance severe sensitivity toward *p*-Cl-Phe. Considering the importance of the RBS region in translation (Barrick et al., 1994; Teramoto et al., 2011; Ravasi et al., 2012; Shi et al., 2018; Opgenorth et al., 2019), RBS*_*mmoX*_* was employed to mediate the translation initiation of PheS^*AG*^, which resulted in profound sensitivity toward *p*-Cl-Phe. Therefore, when developing *pheS* as a CSM in a bacterium, optimizing the RBS region should be considered, and can be easily accomplished using the RBS design software that is now available (Na and Lee, 2010).

## Data Availability Statement

The datasets generated for this study can be found in the GenBank accession nos. WP_047601123.1, WP_041851070.1, WP_010960035.1, ATQ70420.1, WP_017840103.1, CCE22593.1, and WP_033158015.1.

## Author Contributions

XY developed the project idea and revised the manuscript. YL performed most of the experiments, analyzed the data, and prepared the manuscript. XH and PZ did some data analysis and performed some experiments. MC and QH provided consultation for the work and contributed significantly to the preparation of the manuscript. All authors reviewed the manuscript and agreed with the content.

## Conflict of Interest

The authors declare that the research was conducted in the absence of any commercial or financial relationships that could be construed as a potential conflict of interest.
