# Supplementary material for: pheSAG Based Rapid and Efficient Markerless Mutagenesis in Methylotuvimicrobium
Source: Front Microbiol. 2020 Mar 31;11:441. doi: 10.3389/fmicb.2020.00441 (PMC7136838; doi:10.3389/fmicb.2020.00441)
Supplement: Supplementary file 1 [file Table_1.DOCX]

**The** **supplemental materials include one table and one figure.**

TABLE

TABLE S1 Primers used in this study

| primer | sequence（5’-3’） | purpose |
| --- | --- | --- |
| phes1F | aaaagaaaaaccggtcgggtc | Construction of *pheS^AG^-1* |
| phes1R | ttcggcggagggctctgcaaacggaaaataggatg |  |
| phes2F | tattttccgtttgcagagccctccgccgaagtcga |  |
| phes2R | gacccccataccgaaaccgaaacctgaatacatct |  |
| phes3F | tattcaggtttcggtttcggtatgggggtcgagcg |  |
| phes3R | cctgtgtgaatacctctacctaaattgctgcaaaa |  |
| zeoF | cagcaatttaggtagaggtattcacacaggaaaca |  |
| zeoR | tcagtcctgctcctcggcca |  |
| ZPtac1F | ctctgaaatgagctgttgacaattaatcatcggctcgtataatgtgtggtttcgatatttcccattcat | Construction of *pheS^AG^-2* |
| ZPtac2F | ctcgtataatgtgtggttttttcggtaactaacacacaggagaagtcaaatgtcggctactctcgaaga |  |
| ZPtac3F | ctctgaaatgagctgttgacaattaatcatcggctcgtataatgtgtggttttttcggtaactaacac | Construction of *pheS^AG^-3* |
| 485LF-F | ggtgtcgatggcatgctcaa | Construction of 5G- pheS^AG^-1 |
| 485PZ1LF-R | accggtttttcttttcaaggcgaagttgaaggcgc |  |
| 485PZ1-F | ttcaacttcgccttgaaaagaaaaaccggtcgggt |  |
| 485PZ-R | atcgcggagatcgattcagtcctgctcctcggcca |  |
| 485RF-F | gaggagcaggactgaatcgatctccgcgataatct |  |
| 485RF-R | gtgtaacgcatgtaacgcac |  |
| 485PZ23LF-R | cagctcatttcagagcaaggcgaagttgaaggcgc | Construction of 5G- pheS^AG^-2 and 5G- pheS^AG^-3 |
| 485PZ23-F | ttcaacttcgccttgctctgaaatgagctgttgac |  |
| gA1LF-F | attcatgggttgcgagttcg | Construction of 5G-△glgA1 |
| gA1LF-R | aaatccttaatcgagaattaaatttaaaggcagtc |  |
| gA1DR-F | cctttaaatttaattctcgattaaggatttggtcg |  |
| gA1DR-R | cagctcatttcagaggcgaaaggtataatgactaa |  |
| gA1PZ-F | cattatacctttcgcctctgaaatgagctgttgac |  |
| gA1PZ-R | aagaattcgtttcattcagtcctgctcctcggcca |  |
| gA1RF-F | gaggagcaggactgaatgaaacgaattctttttgtc |  |
| YZ1-F | tagctgtagcgctggacga |  |
| YZ1-R | acttcaccaatacgacttca |  |
| MOLF-F | cccgtttagagagtaaggac | Construction of 5G-△smmo |
| MOLF-R | ccaaacagcgacacgaagtg |  |
| MODR-F | ttgtcgcagtaatatttgacttctcctgtgtgtta |  |
| MODR-R | cacaggagaagtcaaatattactgcgacaagctgt |  |
| MOPZ-F | tagcgtagtgtttggctctgaaatgagctgttgac |  |
| MOPZ-R | agcacttattgccattcagtcctgctcctcggcca |  |
| MORF-F | gaggagcaggactgaatggcaataagtgctgcaac |  |
| MORF-R | gtccagaacgcattattcaa |  |
| YZ2-F | ccaaacagcgacacg |  |
| YZ2-R | gatcaagcaaatcgatgcat |  |
| PZ*LF-F | ctctgaaatgagctgttgac | Construction of PZ* cassette |
| PZ*LF-R | cctgtgtgaatacctctatctgaactgttgcagga |  |
| PZ*RF-F | caacagttcagatagaggtattcacacaggaaaca |  |
| PZ*RF-R | tcagtcctgctcctcggcca |  |
| gALF-F | ccttgatgtttacctacccg | Construction of 20Z-△glgA1 |
| gALF-R | aaatccttaatcgagggcagtcaaaattcggttct |  |
| gADR-F | cgaattttgactgccctcgattaaggatttggtcg |  |
| gADR-R | cagctcatttcagagcagtgtctctttaggcactg |  |
| gAZP-F | cctaaagagacactgctctgaaatgagctgttgac |  |
| gAZP-R | aagaattcgtttcattcagtcctgctcctcggcca |  |
| gARF-F | gaggagcaggactgaatgaaacgaattctttttgt |  |
| gA RF-R | ccttattggtttgtttgtga |  |
| YZ3-F | catatcggcgtgccttccga |  |
| YZ3-R | ggtcgtcaaaggacgtcgtg |  |

FIGURE


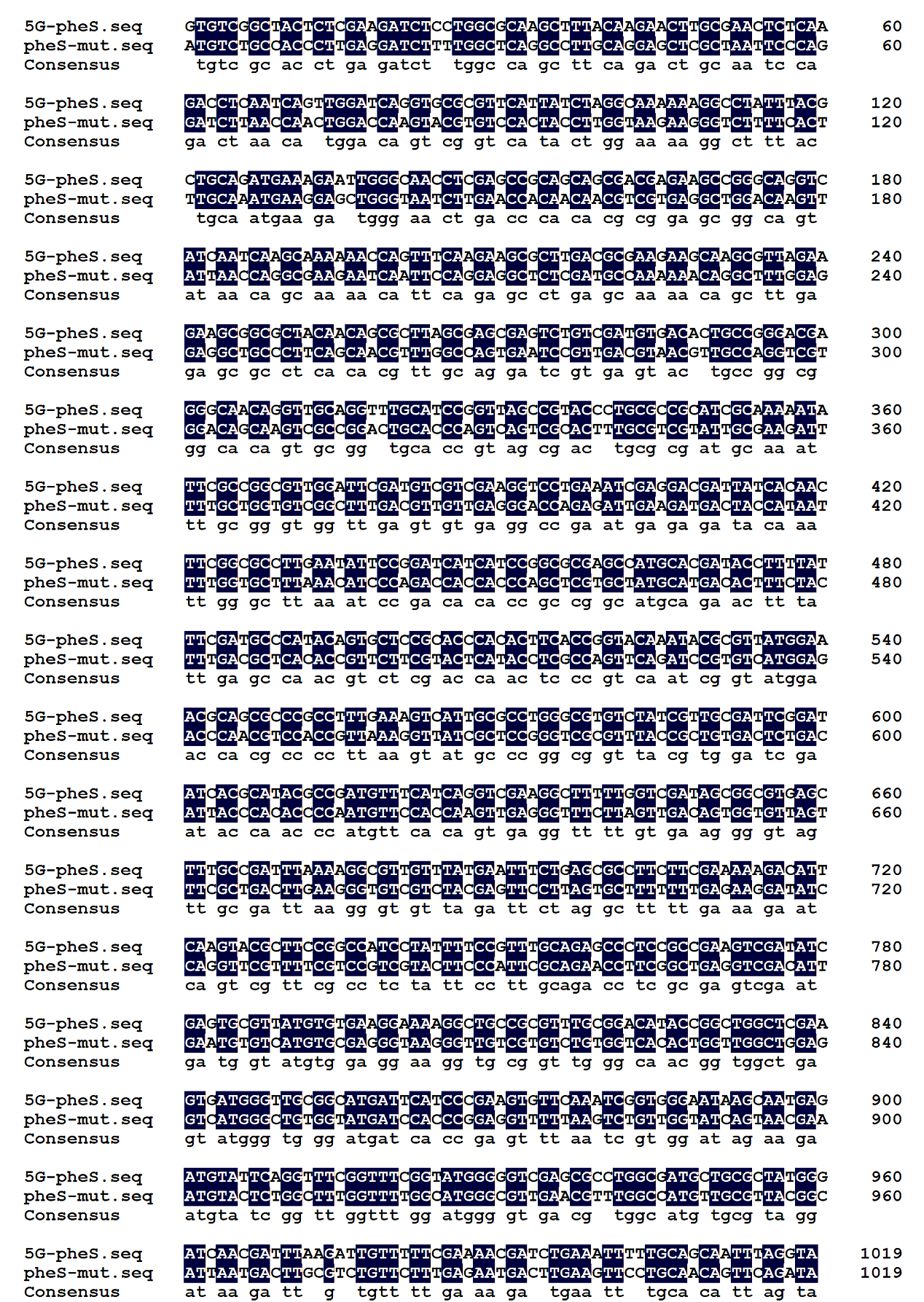


**Fig S1** Artificially synthesized *pheS* gene (*pheS*-mut). The nucleotide sequence of *pheS*-mut was aligned to wild-type *pheS* using Clustal W2.
